# Individualized treatment decision model for inoperable elderly esophageal squamous cell carcinoma based on multi-modal data fusion

**DOI:** 10.1186/s12911-023-02339-5

**Published:** 2023-10-23

**Authors:** Yong Huang, Xiaoyu Huang, Anling Wang, Qiwei Chen, Gong Chen, Jingya Ye, Yaru Wang, Zhihui Qin, Kai Xu

**Affiliations:** 1 Department of Medical Oncology, The Second People’s Hospital of Hefei, Hefei, China; 2 Department of Chinese Integrative Medicine Oncology, The First Affiliated Hospital of Anhui Medical University, Hefei, China; 3 Scholl of Internet, Anhui University, Hefei, China; 4 Department of Radiation Oncology, The First Affiliated Hospital of Anhui Medical University, Hefei, China

**Keywords:** Esophageal squamous cell carcinoma, Inoperable elder patients, Concurrent chemoradiotherapy, Radiotherapy, Individualized treatment decision model

## Abstract

**Background:**

This research aimed to develop a model for individualized treatment decision-making in inoperable elderly patients with esophageal squamous cell carcinoma (ESCC) using machine learning methods and multi-modal data.

**Methods:**

A total of 189 inoperable elderly ESCC patients aged 65 or older who underwent concurrent chemoradiotherapy (CCRT) or radiotherapy (RT) were included. Multi-task learning models were created using machine learning techniques to analyze multi-modal data, including pre-treatment CT images, clinical information, and blood test results. Nomograms were constructed to predict the objective response rate (ORR) and progression-free survival (PFS) for different treatment strategies. Optimal treatment plans were recommended based on the nomograms. Patients were stratified into high-risk and low-risk groups using the nomograms, and survival analysis was performed using Kaplan–Meier curves.

**Results:**

The identified risk factors influencing ORR were histologic grade (HG), T stage and three radiomic features including original shape elongation, first-order skewness and original shape flatness, while risk factors influencing PFS included BMI, HG and three radiomic features including high gray-level run emphasis, first-order minimum and first-order skewness. These risk factors were incorporated into the nomograms as independent predictive factors. PFS was substantially different between the low-risk group (total score ≤ 110) and the high-risk group (total score > 110) according to Kaplan–Meier curves (*P* < 0.05).

**Conclusions:**

The developed predictive models for ORR and PFS in inoperable elderly ESCC patients provide valuable insights for predicting treatment efficacy and prognosis. The nomograms enable personalized treatment decision-making and can guide optimal treatment plans for inoperable elderly ESCC patients.

## Background

Esophageal cancer, a prevalent malignancy, ranks sixth in global cancer mortality rates [1]. Esophageal squamous cell carcinoma (ESCC) is the predominant histological type in Asian countries [2, 3]. Standard treatment for ESCC usually involves preoperative chemotherapy or chemoradiotherapy (CCRT) with planned surgery [4, 5]. However, advanced age or contraindications often render elderly patients unsuitable for surgical treatment. In cases where local advanced esophageal cancer is inoperable, concurrent CCRT is often considered to result in better survival outcomes than radiotherapy (RT) alone [6, 7]. Nonetheless, many elderly patients (≥ 65 years old) may not tolerate CCRT. Utilizing data extracted from the Surveillance, Epidemiology, and End Results (SEER) database, it is discernible that elderly individuals afflicted with esophageal cancer experience favorable outcomes when subjected to RT in comparison to their counterparts who do not receive such intervention, especially when the malignancy is confined to a localized or regional stage [8]. Furthermore, a comprehensive analysis of a nationwide database evinces a prevailing trend wherein RT is more frequently administered to elderly patients [9]. Nevertheless, the extant body of evidence does not conclusively establish the efficacy of CCRT in conjunction with RT as a therapeutic modality offering discernable benefits for elderly ESCC patients. Relevant studies have provided limited evidence regarding the superiority of CCRT over RT [10, 11], emphasizing the importance of carefully selecting treatment strategies for elderly patients with esophageal cancer. The optimal treatment approach (CCRT or RT) for elderly patients with inoperable ESCC remains unclear in clinical practice. As population aging has led to an increasing number of elderly ESCC patients [12, 13], early identification of patients who are at a heightened risk of rapidly progressing to CCRT or RT assumes paramount importance in formulating precise treatment strategies that can lead to an enhanced clinical outcome [14].

In evaluating the probability of an individual patient's progression toward CCRT or RT, prior investigations have predominantly utilized conventional statistical analytical approaches. These studies have centered their attention on clinical parameters, including but not limited to age, gender, TNM clinical staging, and radiation dosage, in order to scrutinize the risk factors linked to diverse therapeutic alternatives [7, 15]. However, these clinical factors in isolation prove inadequate in comprehensively capturing the heterogeneity observed in clinical outcomes. Assessing the individual patient's potential progression probability to CCRT or RT poses a considerable challenge. Preceding the commencement of treatment, patients typically undergo a series of additional assessments, encompassing computed tomography (CT) scans, complete blood count examinations, liver and kidney function evaluations, as well as coagulation function tests. These assessments yield radiomics data derived from CT scans and metabolomics data gleaned from blood analyses, which, when amalgamated with baseline information, constitute a personalized multimodal dataset. Integrating these multimodal data may help to comprehensively assess the risks and survival benefits associated with CCRT and RT in nonsurgical elderly ESCC patients. Notably, the analysis of risk factors and the prediction of survival outcomes among this specific cohort, based on multimodal data, present formidable challenges to conventional statistical methodologies. In this context, machine learning (ML) based approaches [16] emerge as promising avenues, as they possess the capacity to effectively scrutinize multimodal data and unveil intricate linear or nonlinear relationships between risk factors and patient survival outcomes [17]. Within the realm of clinical practice, ML methodologies, as well as deep learning methods [18–20], have already demonstrated their proficiency in the identification of pertinent risk factors and the provision of personalized treatment recommendations [21].

Our motivation is further reinforced by the promise of clinical multimodal ML systems to potentially surpass the performance of unimodal systems, capitalizing on the amalgamation of information from diverse routine data sources [22–24]. In this study, 189 cases of locally advanced elderly ESCC patients who were ineligible for surgical treatment were analyzed. ML methodologies were systematically applied to evaluate multimodal data acquired prior to the initiation of treatment, with a primary objective of discerning pivotal risk factors that exert influence on treatment efficacy and prognosis. Within this study, an innovative model denominated as the Combined Treatment Decision for Efficacy and Prognosis Nomogram (CTDEPN) was developed. This CTDEPN model serves as a predictive tool, enabling the assessment of treatment efficacy and prognosis for distinct therapeutic regimens in inoperable elderly ESCC patients, thereby facilitating the tailored recommendation of optimal treatment strategies.

## Methods

### Patients and assessment

This retrospective study has obtained approval from the Ethics Committee, and it has been granted an exemption from the necessity of acquiring informed consent. A total of 189 cases were retrospectively enrolled from a cohort of inoperable elderly ESCC patients who received either CCRT or RT at the authors' institution between 2013 and 2023. Of these cases, 169 were obtained from the Second People's Hospital of Hefei, subsequently referred to as "Institution 1," while the remaining 20 cases were sourced from the First Affiliated Hospital of Anhui Medical University, hereinafter referred to as "Institution 2″.The inclusion criteria were as follows: 1) age ≥ 65 years; 2) histologically confirmed esophageal squamous cell carcinoma; 3) clinical stage II-IV; 4) performance status (PS) score ≤ 2; 5) absence of any history of tumor or radiation therapy; 6) absence of severe concomitant medical comorbidities; 7) availability of CT imaging data obtained within 2 weeks before the initiation of treatment.

The exclusion criteria consisted of the following: 1) age < 65 years; 2) histologically confirmed non-esophageal squamous cell carcinoma; 3) PS score > 2; 4) presence of concomitant other malignant tumors; 5) presence of severe concomitant medical comorbidities; 6) presence of artifacts, blurriness, errors, or disordered slices in the CT images.

**Fig. 1:**
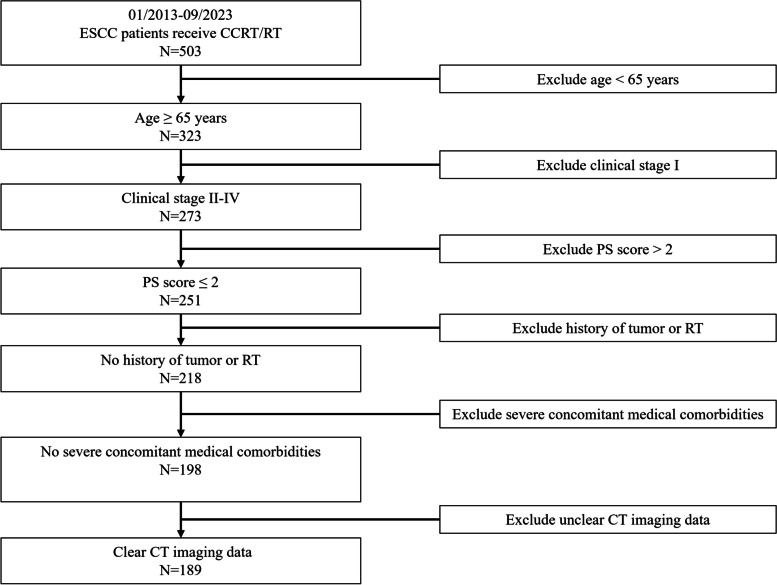
Screening of enrolled cases based on inclusion and exclusion criteria

All participants underwent comprehensive baseline assessments, which encompassed physical examinations, complete blood count, blood chemistry tests, barium meal examination, endoscopic biopsy, pulmonary function tests, electrocardiogram, and CT scans of the neck, chest, and abdomen. The CT images of the patients were acquired utilizing a GE Optima 16-row CT simulator. The CT images were characterized by a resolution of 512 × 512 pixels, a reconstruction layer thickness of 5 mm, and the acquisition sequence employed was the plain CT scan protocol. The data characteristics are highly variable depending from hospital to hospital. Therefore, to prove our model is valid, we extra-validate our model from Institution 2. Patients matched from Institution 1 were partitioned into distinct training and testing sets, while patients from Institution 2 were designated for an additional validation set. Data collection for this study continued until September 2023. The patients’ selection of this study was illustrated in Fig. 1.

### Treatment and follow-up

Regarding RT, all patients underwent intensity-modulated radiation therapy (IMRT), with the primary tumor lesion and involved lymph node regions defined as the gross tumor volume (GTV). The clinical tumor volume (CTV) encompassed a 4–8-mm expansion beyond the GTV in all directions and a superior–inferior extension of 3–4 cm. Throughout the treatment period, complete blood count and blood biochemistry were regularly monitored, and a follow-up investigation was scheduled within 2 months after treatment completion. Patients who underwent follow-up assessment also received routine blood and serum biochemistry tests.

Tumor response was assessed in accordance with the Response Evaluation Criteria in Solid Tumors (RECIST) guidelines (version 1.1) [25], using physical examinations and CT scans. The evaluation of tumor response based on CT scans followed the criteria established by Conroy et al. [26], considering the vertical length and maximum transverse thickness of the tumor. Complete response (CR) was defined as the complete disappearance of the primary tumor area. Partial response (PR) was characterized by a reduction of at least 30% in the sum of the diameters of all measurable target lesions compared to the baseline, sustained for a minimum of 4 weeks.

After treatment completion, patients were subjected to regular follow-up at intervals of 2 months during the first year, 3 months during the second and third years, and subsequently every 6 months. Disease progression was evaluated in accordance with RECIST criteria, considering clinical indicators, imaging examinations, or symptomatic signs.

### Outcome measures and definitions

The primary outcome of this study was progression-free survival (PFS), which was defined as the duration from the initiation of treatment until either tumor progression, death attributable to the tumor, or the last follow-up date. The secondary outcome was the objective response rate (ORR), which represented the proportion of patients who achieved CR or PR two months after treatment completion.

### Data preprocessing

The clinical features recorded for the study included age, sex, height, weight, body mass index (BMI), performance status (PS) score, lesion location, histologic grade (HG), clinical stage, T stage, N stage, radiation dose, pre-treatment blood glucose, hemoglobin, and blood albumin values. The ORR and PFS data of patients were extracted and labeled as the targets. Afterward, the clinical features were subjected to data encoding, data cleaning, normalization, and other preprocessing operations to obtain processed data.

The CT imaging data, acquired from patients prior to treatment, were stored in PNG format, with each image slice possessing dimensions of 512 × 512 pixels. Subsequently, these image slices were imported into the ArcMAP software to delineate the regions of interest (ROI), specifically encompassing the general tumor and lymph node metastasis areas in esophageal cancer. To mitigate inter-gradient variability among evaluators, this study implemented a semi-automatic ROI delineation approach. Deep learning techniques [27] based on an improved spatial pyramid model served as a preliminary tool for generating ROIs, subsequent manual modifications and reviews by doctors are required. The delineated tumor areas underwent further refinement and scrutiny by two seasoned radiation oncologists, each possessing over a decade of experience. Following a consensus agreement on the ROI boundaries, a senior physician with more than 15 years of professional expertise subsequently confirmed the final delineation of the ROIs. To eliminate noise interference and irrelevant areas, all CT image intensity values were truncated to the range of (-200HU, 250HU) [28], followed by normalization using the min–max normalization method. Ultimately, patients from Institution 1 were randomly allocated into the training and testing sets at an 8:2 ratio, while patients from Institution 2 were specifically designated for use as an additional validation set. This approach was implemented to guarantee a representative distribution of cases for both model training and evaluation purposes.

### Features extraction

**Fig. 2:**
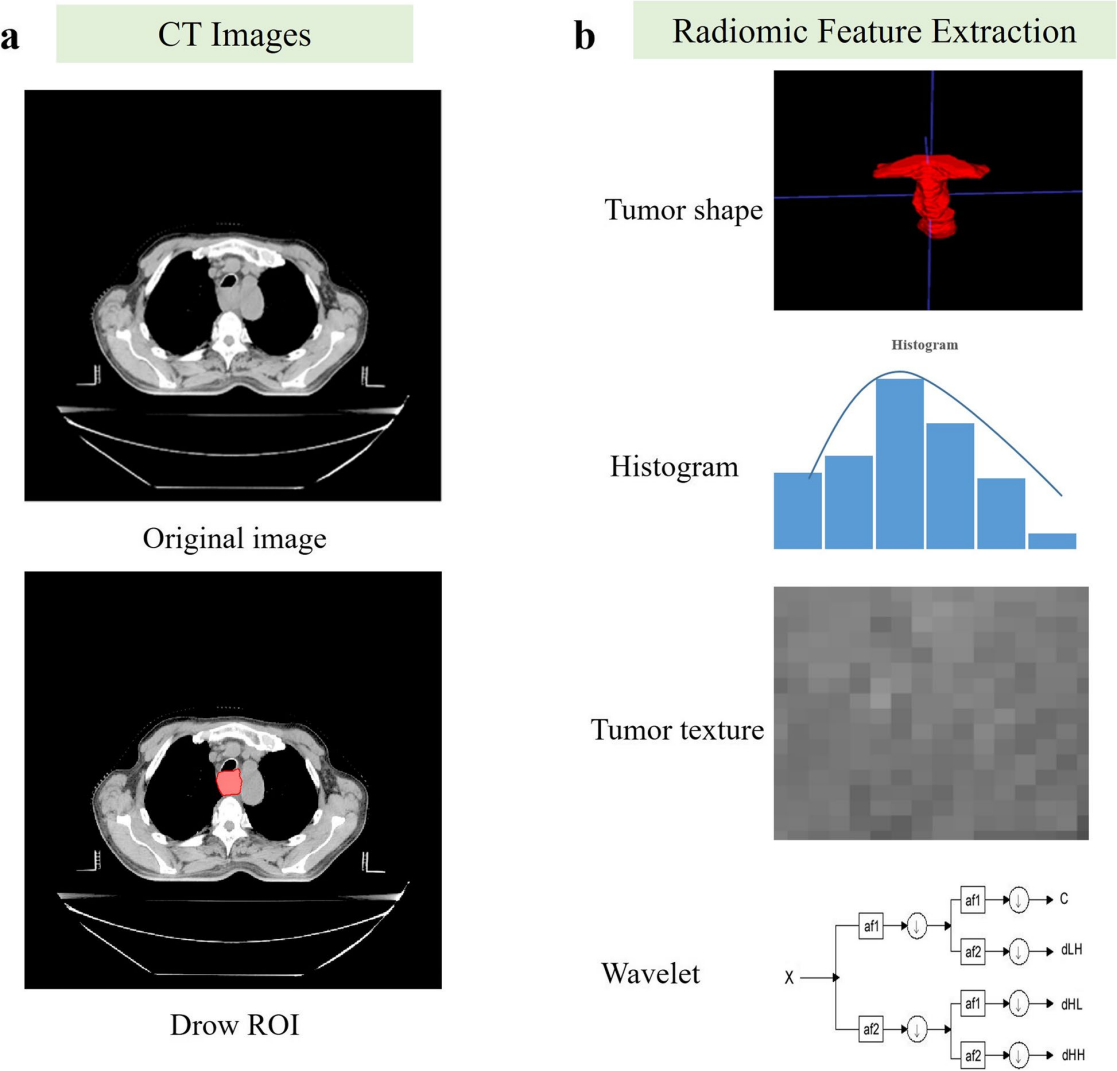
Radiomic feature extraction (**a** The regions of interest (ROI) of tumors were segmented on plain phase CT section; **b** The radiomic feature extraction process)

In this study, we harnessed the power of the PyRadiomics platform [29], a versatile open-source tool, to extract radiomic features from medical images. PyRadiomics, developed in the Python programming language, has gained popularity in the scientific computing community due to its flexibility and can be effortlessly installed on any system. As shown in Fig. 2 and Table 1, the radiomic feature extraction process began by delineating regions of interest (ROIs) in CT images, which served as the foundation for our subsequent analyses. We utilized PyRadiomics to extract four distinct categories of radiomic features: Intensity Features, which involve direct calculations of tumor image grayscale values; Shape Features, typically used to quantify tumor morphology, size, and regularity; Texture Features, employed for quantifying texture patterns and tissue distribution within the tumor, often imperceptible to the human eye; and Wavelet Features, aimed at extracting tumor information across different frequency domains.

**Table 1:** Classification and description of radiomic features to used

Feature Class	First order statistics features	Shape Features	Texture Features	High-order features
Definition	Describe the distribution of voxel intensities within the region of interest (ROI) using common and basic metrics	Descriptors of the two-dimensional size and shape of the ROI	Considers the spatial relationship of pixels. These Features characterize the texture of an image by measuring how often pixel pairs of unique values with a given spatial relationship occur in an image	Obtained by statistical methods after applying filters or mathematical transforms to the images

As the numerical values of quantified radiomic features could exhibit significant variations across different orders of magnitude, and the absolute differences between metric features could be substantial, a normalization step was performed to mitigate the influence of features with disparate dimensions on the weighting of the objective function. To achieve this, the extracted radiomic features were subjected to Z-score standardization [30], following the formula:

1 $$Z=\frac{(x-\mu )}{\sigma }$$

where *z* represented the standardized value, $\mu$ denoted the mean value of the feature in the dataset, and $\sigma$ signified the standard deviation of the values in the sample. This normalization procedure standardized the radiomic features, rendering them comparable and facilitating subsequent analysis and modeling.

The reproducibility of the extracted radiomic features was assessed using the interclass correlation coefficient (ICC) [31]. The features exhibiting an ICC value greater thanexceeding 0.75 were considered to demonstrate favorable reproducibility and robustness, which qualified them for further analysis. The maximum relevance–minimum redundancy (mRMR) method [32] was applied to evaluate the correlation between the features and the risk of recurrence. This method facilitated the identification and elimination of redundant and irrelevant radiomic features, generating relevance-redundancy scores (mRMR scores) for each feature. Subsequently, the features were ranked based on their scores, and the top 10% were selected for further analysis. These selected radiomic features, in conjunction with the preprocessed clinical feature data, were integrated to form the multimodal dataset used for further analysis.

### Risk factors selection

**Fig. 3:**
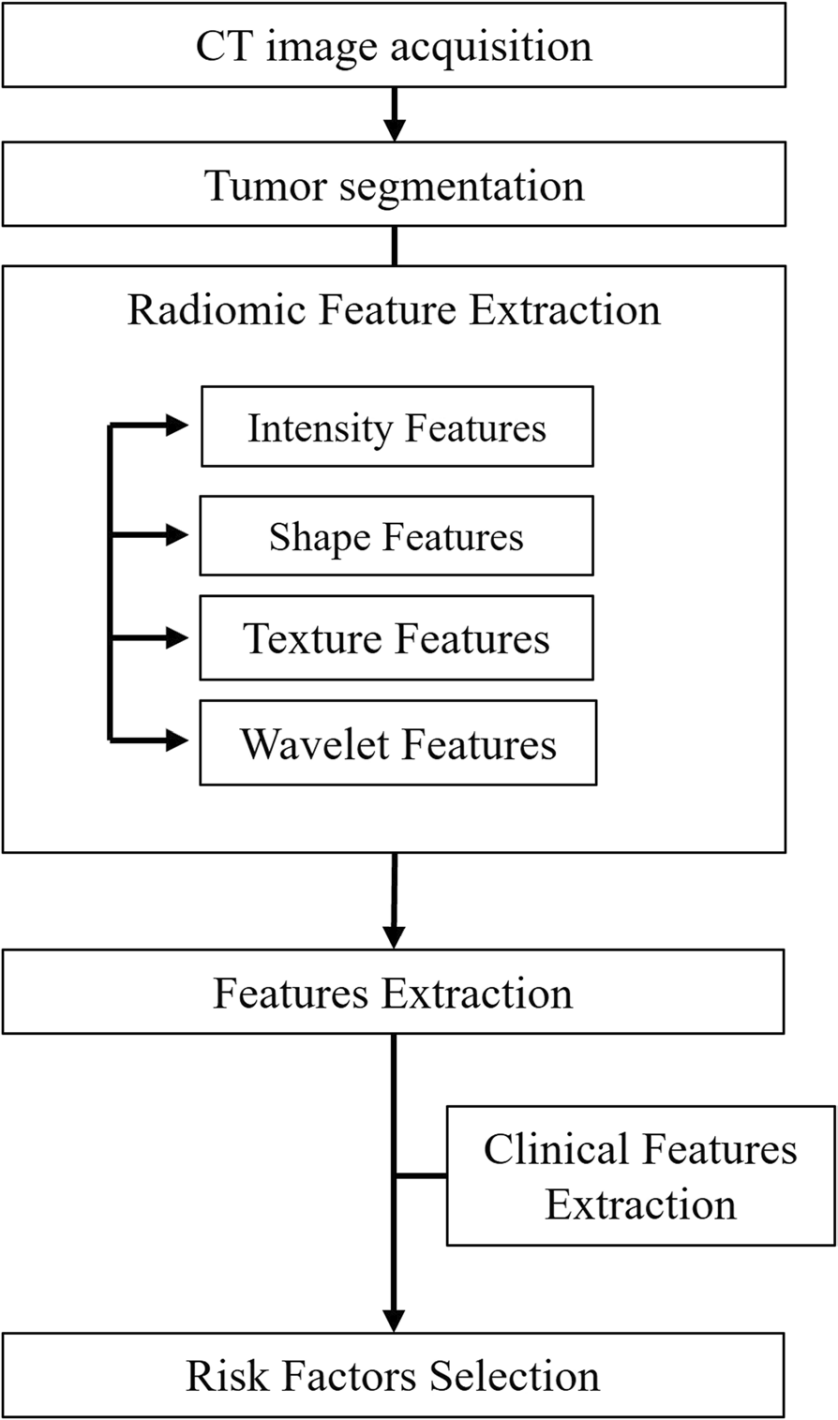
ML method was used to screen and construct risk factors

As shown in Fig. 3, a ML based approach was proposed in this study to identify the risk factors that significantly influence the ORR and PFS of inoperable elderly ESCC patients treated with CCRT or RT. To evaluate the impact of different treatment strategies on prognosis, three distinct cohorts were established within the training set: the overall patient cohort, the CCRT-treated patient cohort, and the RT-treated patient cohort.

Risk factor extraction is an iterative process which is controlled by the choosing of subsets and its validation. Firstly, a premise has been proposed that every single feature can be identified as a subset which will be sorted by performance score. Then, with the instruction of sorted result, the best subset is expanded by adding one feature from high to low until the current selected features’ performance score becomes flatted. For each cohort, the risk factors influencing the ORR and PFS were extracted separately using specific methods, as outlined below:

First, comprehensive feature classification models were developed for inoperable elderly ESCC patients’ post-treatment to predict both the response and progression of ESCC. By employing the Relief feature selection algorithm [33], diverse sets of potential risk factors associated with ESCC response and progression were obtained from the multimodal data. Significant feature sets pertaining to ESCC response and progression were extracted based on the alternative feature sets, and prediction models were established to assess treatment efficacy and prognosis of ESCC, utilizing the extracted risk factors.

Second, risk factor extraction and survival prediction models were developed to assess the impact of ORR and PFS in inoperable elderly ESCC patients’ post-treatment. Comprehensive feature regression models were constructed to investigate the associations between treatment outcomes and various features in the patients. Utilizing these comprehensive feature regression models, surrogate feature sets for ORR and PFS following treatment in the patients were generated. Subsequently, a set of risk factors for ORR and PFS after treatment in inoperable elderly ESCC patients were identified based on the surrogate feature sets. Prediction models for ORR and PFS in inoperable elderly ESCC patients after treatment were established for the three cohorts, leveraging the extracted risk factors.

Based on the difference of target tasks, Relief was used to the classification of ORR while Extra Trees was chosen for the regression of PFS. In addition, for enhancing the model generalization, coefficient of determination R-squared was also used to valid the feature set. There is also an unavoidable challenge that is the uneven distribution of data in such specific task. Therefore, in the step of data pre-processing, SMOTE was adapted to balance the number of samples. All ML experiments were implemented by Python Scikit-Learn package [34], which a simple and efficient tool for predictive data analysis and data is mining.

### Model evaluation

The accuracy of the PFS prediction model were evaluated using the coefficient of determination (R^2^ score), which measured the proportion of the variance in the dependent variable that could be explained by the independent variables. A higher R^2^ score closer to 1 indicated a better fit of the model, while a score of zero or negative indicated poor performance of the model on the dataset. Additionally, the performance of the ORR prediction model and PFS prediction model were evaluated using Receiver Operating Characteristic (ROC) curves and the Area Under the Curves (AUCs). The AUCs ranged from 0.50 to 1.0, with a higher AUC indicating better model discriminative ability. The Hanley & McNeil method [35] was furtherly employed to assess the statistical significance of the difference between our model's AUC and the theoretical random AUC of 0.5. A *p*-value less than 0.05 indicates a significant difference between them, which means this model has better discriminative ability of the model. Calibration curves were used to assess the deviation between the actual and expected outcomes. A calibration curve closer to the diagonal line indicated higher predictive accuracy.

### CTDEPN construction

The CTDEPN was constructed by extracting independent risk factors using the best-performing ORR and PFS prediction models. Risk factors influencing ORR and PFS were extracted from the multimodal data for each of the three cohorts. The relationship between the common risk factors for ORR, the common risk factors for PFS, and different treatment methods within the training set was evaluated. Utilizing CTDEPN, predictions for ORR and PFS were calculated for each patient receiving different treatment regimens, enabling personalized treatment recommendations based on the observed differences in ORR and PFS. The optimal cutoff value for the total score was determined using CTDEPN, leading to the categorization of patients into high-risk and low-risk groups. Survival curves were generated using the K–M method to illustrate the outcomes of the two groups. The log-rank test was used to calculate the *P* value for hazard ratio (HR) estimation, with significance defined as a two-sided *P* value of < 0.05.

## Results

### Patients and characteristics

Baseline characteristics of patients in training set, testing set, and extra validation set were presented in Table 2. No discernable distinctions were identified between the three sets in relation to various demographic and clinical parameters, including age, gender, body mass index (BMI), performance status (PS) score, overall stage, lesion location, histologic grade, T-stage, N-stage, clinical stage, radiation dosage, and treatment regimens.

**Table 2:** Comparison of baseline characteristics in training, testing and extra validation set

Characteristics	Training set	Testing set	Extra Validation set	*P*
	(*n* = 135), %	(*n* = 34), %	(*n* = 20), %	
Age (years)				0.577
≤ 75	78(57.8)	20(58.8)	9(45.0)	
> 75	57(42.2)	14(41.2)	11(55.0)	
Sex				0.873
Male	96(71.1)	24(70.6)	13(65.0)	
female	39(28.9)	10(29.4)	7(35.0)	
BMI (kg/m^2^)				0.574
< 18	29(21.5)	7(20.6)	1(5.0)	
18 ≤ X < 24	87(64.4)	22(64.7)	16(80.0)	
24 ≤ X < 28	16(11.9)	4(11.8)	2(10.0)	
≥ 28	3(2.2)	1(2.9)	1(5.0)	
PS score	(*n* = 135), %	(*n* = 34), %		0.686
0	5(3.7)	2(5.9)	2(10.0)	
1	120(88.9)	30(88.2)	17(85.0)	
2	10(7.4)	2(5.9)	1(5.0)	
Lesion location				0.926
Upper	29(21.5)	8(23.5)	4(20.0)	
Middle	81(60.0)	20(58.8)	14(70.0)	
Distal	25(18.5)	6(17.7)	2(10.0)	
Histologic grade				0.928
Well differentiated	10(7.4)	3(8.8)	1(5.0)	
Moderately differentiated	33(24.4)	9(26.5)	3(15.0)	
Poorly differentiated	17(12.6)	4(11.7)	4(20.0)	
Unknown	75(55.6)	18(53.0)	12(60.0)	
T stage				0.445
T3	53(39.2)	14(41.2)	5(25.0)	
T4	82(60.8)	20(58.8)	15(75.0)	
N stage				0.751
N0	64(47.4)	16(47.1)	11(55.0)	
N1	53(39.3)	13(38.2)	5(25.0)	
N2	18(13.3)	5(14.7)	4(20.0)	
Clinical stage				0.837
stage II	28(20.7)	7(20.6)	3(15.0)	
stage III	59(43.7)	15(44.1)	7(35.0)	
stage IV	48(35.6)	12(35.3)	10(50.0)	
Radiation dose (Gy)				0.565
< 60	85(62.9)	21(61.8)	10(50.0)	
≥ 60	50(37.1)	13(38.2)	10(50.0)	
Treatment regimens				0.128
CCRT	37(27.4)	10(29.4)	10(50.0)	
RT	98(72.6)	24(70.6)	10(50.0)	

### Curves of calibration and validation

**Fig. 4:**
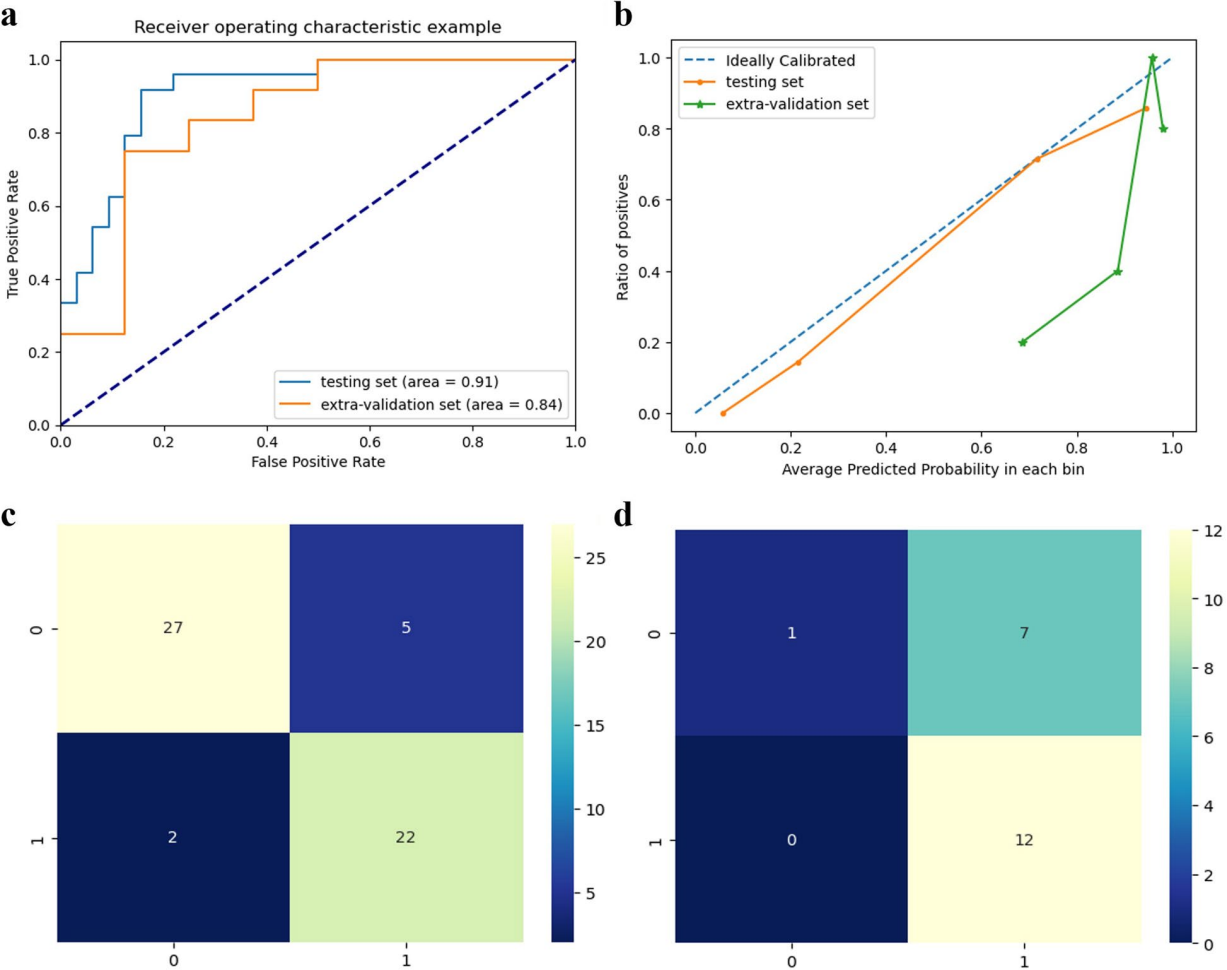
ROC and calibration curves of prediction model in elder ESCC patients (**a**: ROC curves; **b**: calibration curves; **c**: confusion matrix in testing set; **d**: confusion matrix in extra validation set)

Multiple prediction models were constructed using ML techniques, and the prediction model with the best performance were chosen. The ROC and calibration curves of the prediction model were depicted in Fig. 4. In the testing set, the prediction model exhibited an AUC of 0.91, while in the extra validation set, the prediction model demonstrated an AUC of 0.84, indicating the favorable predictive performance. As shown in Fig. 4c, d, in our testing set, we have 22 true positive (TP) examples, 27 true negatives (TN), 2 false positives (FP), and 5 false negatives (FN). In our extra validation set, we have 12 TP examples, 1 TN, 0 FP, and 7 FN. The Hanley & McNeil method demonstrated a statistically significant difference between our model's AUC and the expected random AUC of 0.5, with a calculated *p*-value of 0.02. Through the application of ML method, a comprehensive analysis was conducted on the multimodal data of all patients in the training set of the ORR and PFS prediction models, as well as the cohorts of patients treated with CCRT and RT. Common risk factors influencing ORR and PFS were extracted from each of these three sets. These risk factors were ranked based on their respective influence weights, and the top 5 factors were selected as the ultimate determinants of ORR and PFS.

### CTDEPN performance

In this study, a comprehensive set of 56 features was extracted from the CT images. Among these, three specific features including original shape elongation (OSE), first-order skewness (FOS), and original shape flatness (OSF) demonstrated the most robust associations with the ORR within the training dataset. Similarly, within the training dataset, another trio of features including high gray-level run emphasis (HGLRE), first-order minimum (FOM), and FOS exhibited the strongest associations with PFS. Among them, OSE quantified the shape of an object, with values close to 1 indicating circularity. FOS measured the skewness direction of a data distribution, with positive values indicating right-skew and negative values indicating left-skew. OSF quantified the flatness of an object, with larger values indicating more flatness. HGLRE was a feature that highlights regions with high gray-level continuity in images, facilitating texture analysis. Areas with irregular or heterogeneous structures might have lower HGLRE. FOM measured the minimum value within a dataset [36]. The risk factors influencing ORR in inoperable elderly ESCC patients selected through ML method were HG, T stage, OSE, FOS and OSF, while risk factors influencing PFS included BMI, HG, HGLRE, FOM and FOS. The R^2^ scores of the selected risk factors for the PFS prediction model were shown in Table 3. These risk factors were utilized in the subsequent construction of CTDEPN.

**Table 3:** Coefficients of different risk factors

Risk factor	R^2^ score
HGLRE	0.697179
BMI	0.736510
FOM	0.960427
FOS	0.951633
HG	0.947236

**Fig. 5:**
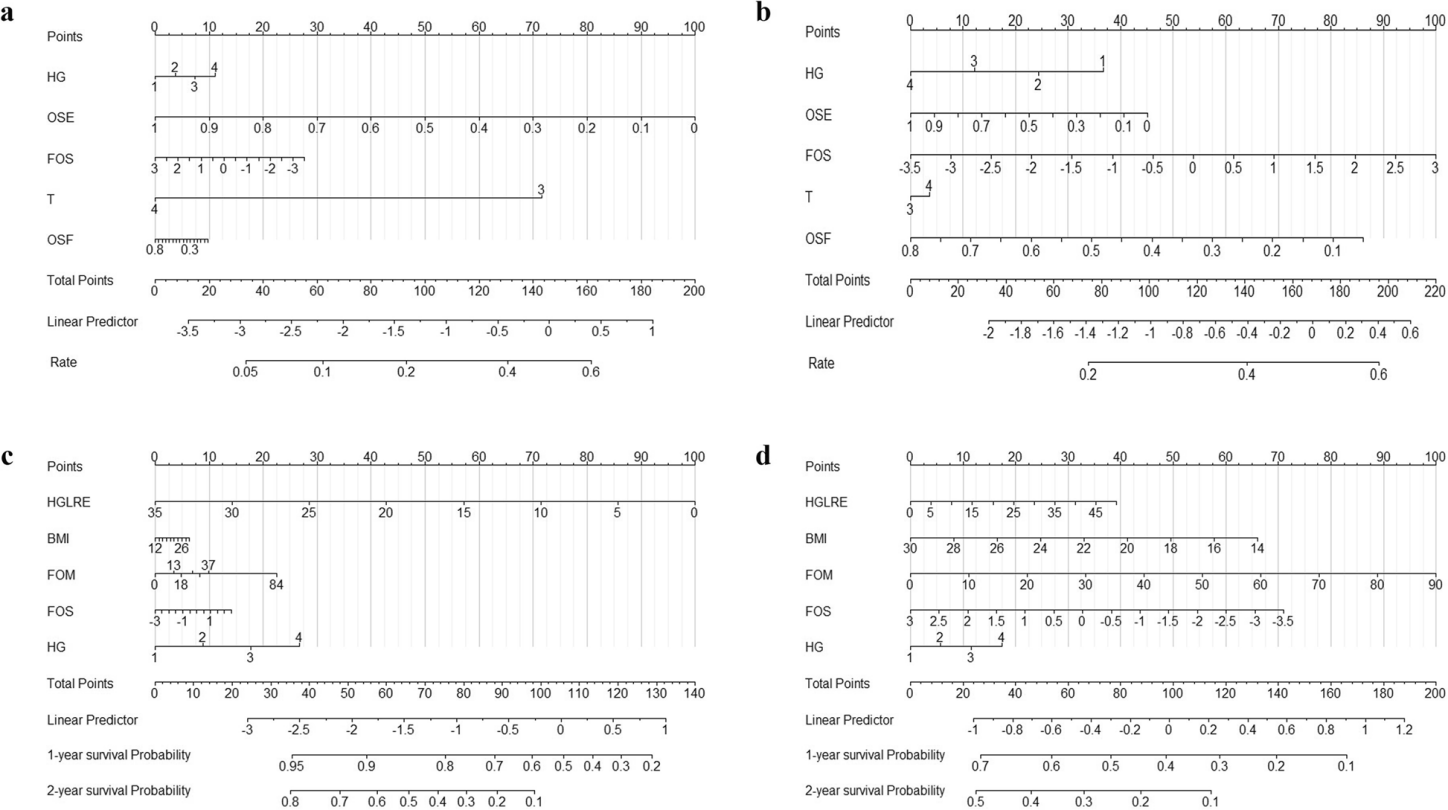
Combined Treatment Decision for Efficacy and Prognosis Nomogram (**a**: CCRT nomogram for ORR; **b**: RT nomogram for ORR; **c**: CCRT nomogram for PFS; **d**: RT nomogram for PFS)

CTDEPN comprised two components: the ORR prediction model and the PFS prediction model. The extracted risk factors were assigned scoring values for the two treatment options, CCRT and RT, respectively. CTDPEN was shown in Fig. 5, where it could be observed that the same risk factors are assigned different values when applying CCRT or RT. Notably, the total score, as well as the ORR, 1-year PFS rate, and 2-year PFS rate, exhibited variations. Through comprehensive calculations, the model recommended treatment options associated with higher ORR and improved 1-year and 2-year PFS rates. When a previously untreated patient is confronted with the decision of opting for CCRT or RT, it becomes possible to integrate the patient's clinical data and CT images into the model. This integration enables the estimation of the 1-year and 2-year Progression-Free Survival (PFS) rates following the administration of CCRT and RT treatments, respectively. Subsequently, the selection of the treatment modality associated with a superior PFS rate can be recommended as the preferred course of action for the patient. For instance, consider an elderly patient with untreated moderately differentiated ESCC, T stage 3, BMI of 20, OSE of 0.5, FOS of 1, OSF of 0.3, HGLRE of 25, and FOM of 40. If the patient undergoes CCRT, the total score for ORR is 170, resulting in a 52% probability of ORR. The total score for PFS is 128, corresponding to a 1-year PFS rate of 25%. On the other hand, if the patient undergoes RT, the total ORR score is 140, with a 47% probability of ORR. The total PFS score is 61, leading to a 1-year PFS rate of 87%. Based on these calculations, CTDEPN recommends RT treatment for this patient.

### Curves of survival

**Fig. 6:**
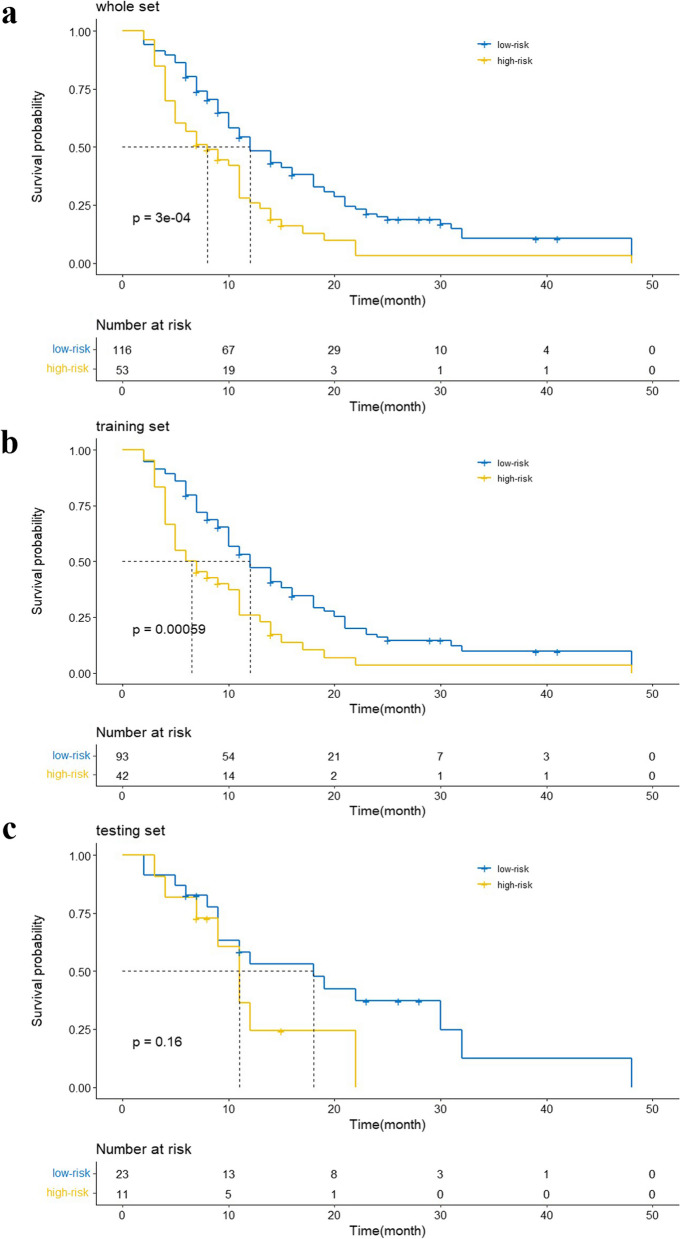
Kaplan–Meier curves of patients in low and high-risk groups. (**a**: whole set; **b**: training set; **c**: testing set)

According to the total score assigned to each patient by the CTDEPN, the patients were categorized into two groups, namely, the high-risk group (total score > 110) and the low-risk group (total score ≤ 110). K–M survival curves depicting the outcomes of the high-risk and low-risk groups are presented in Fig. 6 for the whole set, training set, and testing set. Between the two distinct risk categories, there was a sizable variation in PFS in the whole set and the training set (Fig. 6a, b). In the testing set, the median PFS in the low-risk group was longer, yet, the difference appeared negligible (Fig. 6c).

## Discussion

Esophageal cancer is a prevalent malignancy, the incidence of which has been steadily rising in the elderly population. While the Radiation Therapy Oncology Group (RTOG) 85–01 trial has demonstrated better prognoses in esophageal cancer patients undergoing definitive chemoradiotherapy than in those treated with radiation therapy alone [37], treatment decisions should not only take into account patients’ age, but also their functional status, risk of treatment-related morbidities, life expectancy, and patients’ preference [38, 39]. Careful selection between CCRT and RT remains vital for elderly individuals with unresected locally advanced ESCC [40]. Considering the growing aging population [41], it is essential to identify the risk factors that impact the ORR and PFS rate using multi-modal data and assess the association between these risk factors and different treatment approaches. In this study, we developed a nomogram, the CTDEPN, to aid in prognostic prediction and treatment decision-making for inoperable elderly ESCC patients. The CTDEPN comprises two components: ORR prediction and PFS prediction. Here, by assigning scores based on the risk factors associated with CCRT and RT, the CTDEPN enabled the prediction of patient outcomes under different treatment regimens and provided recommendations for optimal treatment approaches.

A previous study investigating machine-learning methods for exploring prognostic risk factors in esophageal cancer focused on extracting mRNA transcriptomic data from public databases, such as The Cancer Genome Atlas (TCGA), to assess the predictive capability of the models for ORR or PFS [42]. Another study aimed to identify novel biomarkers that could predict treatment outcomes [43]. However, these studies were limited to patients undergoing CCRT for esophageal cancer and did not encompass patients receiving different treatment regimens or specifically focus on elderly patients. Given the impact of treatment approaches on clinical outcomes, it is imperative to consider treatment-related factors when analyzing prognosis. Nevertheless, these studies could not predict patient prognosis under various treatment regimens. In contrast, the CTDEPN, which integrates ORR prediction and PFS prediction, enabled the personalized assessment of ORR and PFS in inoperable elderly ESCC patients under diverse treatment regimens, such as CCRT and RT. Hence, the CTDEPN could provide informed recommendations regarding the optimal treatment approach.

The accurate prediction of ORR and PFS in inoperable elderly ESCC patients is of paramount importance. Accurate prediction models enable the formulation of personalized treatment plans in clinical practice. In this study, multiple prediction models were developed using ML methods. Among these models, the ORR prediction model and PFS prediction model, which exhibited the highest area under the curve (AUC) and calibration curves closest to the diagonal line, were selected. The AUC of the ORR prediction model is 0.80, while the AUC of the PFS prediction model is 0.73, indicating good predictive performance. The difference between the data set’s samples and the model’s predictions, or R^2^ score, is one of the performance evaluation metrics for regression-based ML models. A perfect model would have an R^2^ score of 1, whereas a score of zero or lower indicates poor performance on unseen datasets. In the case of the PFS prediction model, the R^2^ score was calculated as 0.947236, indicating its high predictive accuracy.

In this study, the ORR and PFS prediction models were constructed to analyze patients' multimodal data comprehensively and identify the risk factors influencing ORR and PFS. CTDEPN was developed to predict patients' ORR and 1-year, 2-year PFS rates. The analysis revealed that the risk factors influencing ORR were HG, T stage and three radiomic features including OSE, FOS and OSF, while risk factors influencing PFS included BMI, HG and three radiomic features including HGLRE, FOM and FOS. In the study by Liu et al. [44], PFS in ESCC patients aged ≥ 65 was associated with radiation duration, local recurrence, and disease-related death. The results of this study differ from those of Liu et al., possibly due to the fact that their study only analyzed clinical features and had a relatively small sample size. Interestingly, our study did not reveal age to be a significant prognostic risk factor, consistent with previous research [45]. However, caution is still recommended when treating elderly patients based on other studies [46, 47].

In CTDEPN, the assignment of scores for the same risk factors differs depending on whether CCRT or RT is applied, leading to varying total scores and ORR, 1-year, and 2-year PFS rates. CTDEPN calculates and suggests the treatment option that yields higher ORR and 1-year/2-year PFS rates. Risk factors were assigned distinct scores within the monochromatic chart of the prediction model, resulting in varying total scores and the subsequent prediction of 1-year and 2-year PFS rates for patients. When a newly diagnosed and untreated patient is faced with the decision between CCRT and RT, their clinical data and CT images can be integrated into the prediction model. This integration facilitates the estimation of 1-year and 2-year PFS rates following CCRT and RT treatments, respectively. The treatment modality associated with a superior PFS rate can thus be recommended as the preferred course of action for the patient. It is worth noting that there exists empirical evidence supporting the recommendation of CCRT for inoperable elderly ESCC patients, particularly those exhibiting low BMI, higher HG, lower HGLRE, and larger FOM and FOS values.

All patients were categorized into two groups according to the total score obtained from the CTDEPN, namely, the high-risk group (total score > 110) and the low-risk group (total score ≤ 110). K–M survival curves were generated for the high-risk and low-risk groups using the overall dataset, training set, and testing set. A noticeable difference was observed in both the overall dataset and the training set, with the low-risk group exhibiting a significantly higher median PFS rate than the high-risk group. In the testing set, the low-risk group also displayed a higher median PFS rate than the high-risk group, although with a less pronounced difference. These findings align with those of previous studies [48]. However, contrasting results have been reported in other studies where elderly ESCC patients, regardless of receiving CCRT or RT treatment, achieved longer median PFS durations [11]. This disparity may be attributed to the inclusion of early-stage patients with clinical stage I disease in those studies, while most of the patients in our study were at stages III and IV.

Insufficient evidence exists to substantiate the application of CCRT or RT in the treatment of inoperable elderly ESCC patients, and the therapeutic significance of these modalities remains unclear. In the context of our study, it was observed that patients exhibiting lower BMI, lower HG, reduced HGLRE, larger FOM, and a greater FOS were more likely to be considered suitable candidates for CCRT. Nonetheless, it is imperative to exercise prudence and careful clinical judgment, as the potential risks associated with these therapies may outweigh the anticipated benefits depending on the individual patient's condition. Therefore, taking into account the delicate balance between risk and benefit, the strength of available evidence, and patient preferences, our findings suggest that there is only weak evidence to support the recommendation of CCRT for inoperable elderly ESCC patients who exhibit characteristics such as low BMI, low HG grade, lower HGLRE, larger FOM, and increased FOS.

Despite certain limitations, such as the retrospective nature of this study and the lack of external validation for the created models, the CTDEPN appears to have the best internal validation. Moreover, as the data were solely obtained from the authors’ institution, the findings may not represent patients from other regions, and geographical variations may be present. Further research is necessary to confirm the findings of this study through external validation and the inclusion of data from a more diverse range of sources.

## Conclusions

In the present study, a multitask nomogram that can noninvasively predict the efficacy and prognosis of different treatment options for elderly inoperable ESCC patients using their multi-modal data before treatment was developed and validated. The nomogram can recommend the optimal treatment regimen according to the prediction results. The CTDEPN is a noninvasive and valuable tool that can facilitate personalized treatment and optimize management for elderly inoperable ESCC patients. Future research in the domain of machine learning-related models holds the potential to enable more extensive prospective investigations aimed at validating the model's performance and augmenting its practical applicability.

## Data Availability

All data generated or analyzed during this study are included in this article. Further inquiries can be directed to the corresponding authors.
